# Complement in Pancreatic Disease—Perpetrator or Savior?

**DOI:** 10.3389/fimmu.2017.00015

**Published:** 2017-01-17

**Authors:** Lucas Bettac, Stephanie Denk, Thomas Seufferlein, Markus Huber-Lang

**Affiliations:** ^1^Department of Internal Medicine I, University Hospital of Ulm, Ulm, Germany; ^2^Department of Orthopedic Trauma, Hand, Plastic and Reconstructive Surgery, University Hospital of Ulm, Ulm, Germany

**Keywords:** pancreas, complement, pancreatitis, acinar cells, multiple organ failure, pancreatic ductal adenocarcinomas

## Abstract

The complement system is a major pillar of the humoral innate immune system. As a first line of defense against pathogens, it mediates early inflammatory response and links different branches of humoral and cellular immunity. Disorders affecting the exocrine pancreas, such as acute pancreatitis, potentially lead to a life-threatening systemic inflammatory response with aberrant activation of complement and coagulation cascades. Pancreatic proteases can activate key effectors of the complement system, which in turn drive local and systemic inflammation. Beyond that, the extent of pancreas–complement interaction covers complex pro- and anti-inflammatory mechanisms, which to this day remain to be fully elucidated. This review provides a comprehensive overview of the pathophysiological role of complement in diseases of the exocrine pancreas, based on existing experimental and clinical data. Participation of complement in acute and chronic pancreatitis is addressed, as well as its role in tumor immunology. Therapeutic strategies targeting complement in these diseases have long been proposed but have not yet arrived in the clinical setting.

## Introduction

The pancreas is a central and highly interconnected organ, anatomically as well as metabolically. In local and systemic inflammation, the pancreas is critically involved both as target and effector. Moreover, the pancreas seems to have a so far underappreciated major impact in triggering, development, and progression of multiple organ dysfunction syndrome (MODS) (1). Clinically and experimentally, manifold interactions of the pancreas with the innate and adaptive immune system have been described (2). On a cellular level, leukocytes seem to be significantly affected by pancreatic proteases and mediators that in turn promote local pancreatic (3–5) and systemic inflammation (2). In critically ill patients, e.g., after tissue trauma–hemorrhage, during systemic inflammation, infection, or sepsis, both danger- and pathogen-associated molecular patterns can induce an excessive activation of the complement system (6). The generated complement activation products are potent drivers of local and systemic inflammation and major contributors to MODS (7, 8). However, the pathophysiological interactions of the complement cascade with the pancreas [e.g., during acute pancreatitis (AP)] are rather complex, are partially controversial, and might have been underestimated so far (9). It is even more surprising that the physiological role of complement concerning the exocrine function of the pancreas has barely been investigated.

The aim of this comprehensive review is to provide an up-to-date insight into the interactions of complement with the exocrine pancreas based on experimental and clinical data. The review will neither cover the various functions of complement in regard to the endocrine pancreas (10–14) nor address the potential harmful role of complement in the context of pancreas whole organ and islet transplantation (15–17). The focus of the present review is rather set on various important pancreatic diseases in the light of complement action and regulation.

## Complement as a Main Pillar of Innate Immunity

The cellular and humoral defense systems of innate immunity are crucially involved in inducing and mounting local inflammatory processes, systemic inflammation, and remote organ injury. Main pathophysiological drivers of the inflammatory response are the complement and coagulation cascasdes, which are highly interactive and can also be considered parts of a global serine protease system (18). It is well established that severe pancreatic tissue damage and pancreatitis is associated with activation of the coagulation system, often resulting in development of coagulopathy. In contrast, activation of the complement system and development of complementopathy in the setting of severe pancreatic diseases has not been conclusively investigated so far.

**Figure 1:**
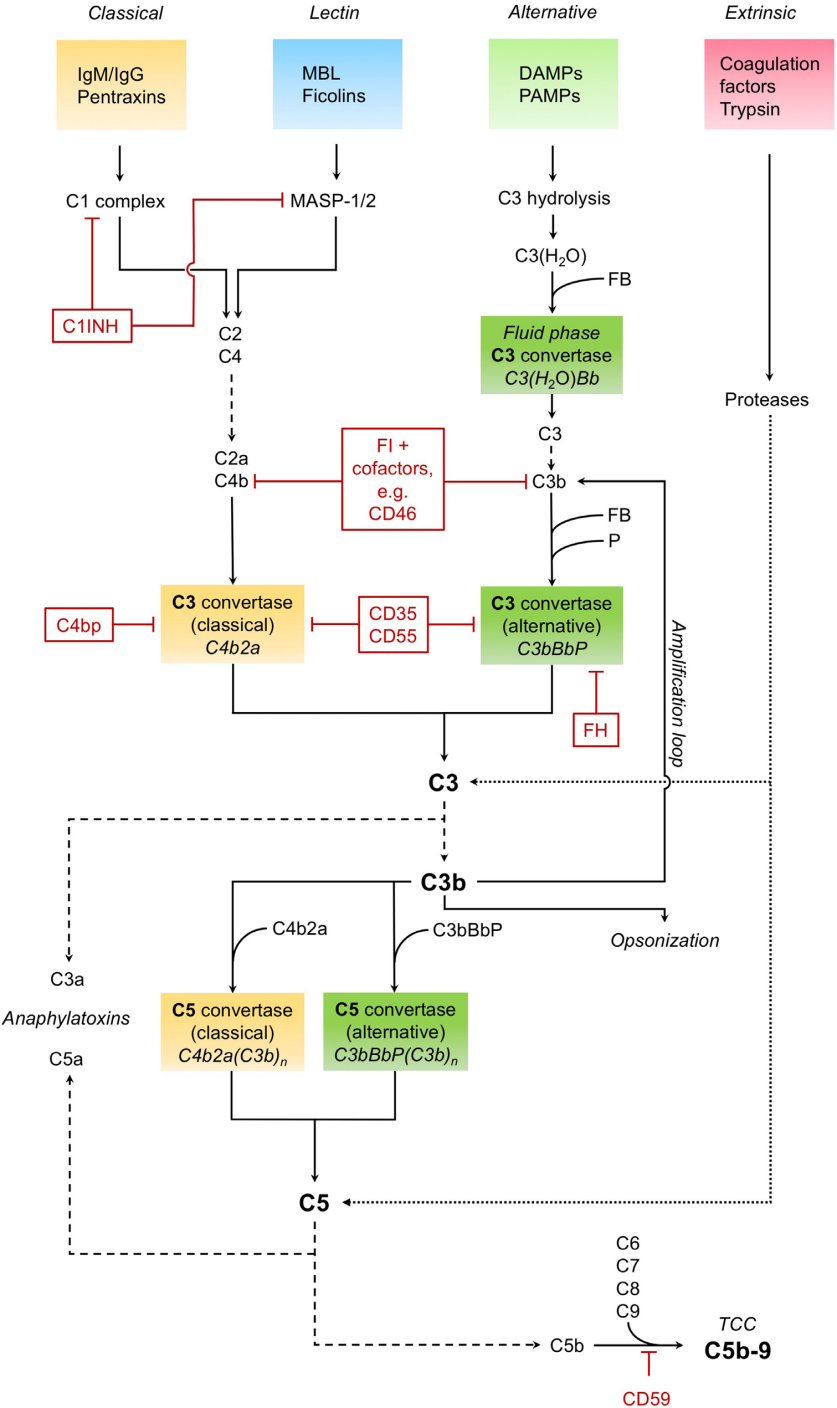
**Complement activation**. The *classical pathway* recognizes immunoglobulins (IgM/IgG) and pentraxins (such as C-reactive protein) and forms and activates a C1 complex, leading to the cleavage of C4 and C2. The cleavage products C4b and C2a form the C3 convertase C4b2a of the classical pathway, which in turn cleaves the central complement component C3 into the anaphylatoxin C3a and opsonin C3b. The C3 convertase can also be formed and activated by the *lectin pathway*, in which mannose-binding lectin (MBL) and ficolins recognize pathogenic patterns, leading to the activation of MBL-associated serine proteases MASP-1 and MASP-2. The *alternative pathway*, triggered by various danger- and pathogen-associated molecular patterns (DAMPS and PAMPS, respectively) on necrotic, apoptotic, and foreign surfaces, involves hydrolisation of C3 and cleavage by other complement factors. Binding of factor B (FB) to C3(H_2_O) results in the formation of an alternative fluid phase C3 convertase. C3 is cleaved, and C3b binds FB to form the surface-bound main C3 convertase of the alternative pathway, which is stabilized by properdin (P) and can in turn induce all functions of complement (C3bBbP). The alternative pathway also constitutes an amplification loop for the complement cascade, with C3b as a feedback effector. Besides enhancing the complement response, as an opsonin, C3b is also essential for the clearance of pathogens and tissue debris. Downstream of the converging activation pathways, the C3 convertases of both classical and alternative pathways bind C3b and form the respective C5 convertases. Those cleave C5, producing the potent anaphylatoxin C5a and C5b. The latter, together with several other complement factors (C6, C7, C8, and C9), forms the terminal complement complex (TCC), also known as membrane attack complex. This C5b–9 complex induces both pro- and anti-inflammatory cell signaling pathways and is also capable to form a lytic pore. Cleavage and activation of central complement components is also possible by proteases typically not associated with the complement system, such as trypsin—effectively constituting a fourth, non-canonical *extrinsic protease pathway* of complement activation (targets shown exemplarily). Important regulators of the complement cascade (CRegs) are C1 inhibitor (C1INH), factor H (FH), and membrane inhibitor of reactive lysis (CD59). Several CRegs control the formation, activity, and degradation of C3 convertase, which can be classical, alternative, or both. CD35, C4bp, and FH, shown here acting on the convertases themselves, can also function as cofactors for FI-mediated cleavage of upstream components C3b or C4b. CD35 = complement receptor 1 (CR1); CD46 = membrane cofactor protein (MCP); CD55 = decay accelerating factor (DAF); FI, factor I; C4bp, C4-binding protein.

The complement system as a key immune surveillance system (19) consists of more than 30 complement components, which can be activated by different pathways. Complement activation *via* classical, alternative, and lectin pathways has been described in great detail before. The main drivers of these pathways are outlined in Figure 1.

It is noteworthy that various complement factors can be activated in a non-canonical manner by an “extrinsic protease pathway” (19), consisting of serine proteases of the coagulation and fibrinolytic system (e.g., thrombin, plasmin, and factor VII-activating serine protease) (20, 21). Furthermore, other proteases such as granzyme B (22) and trypsin (23) are capable of cleaving and activating central complement components, leading to generation of the anaphylatoxins C3a and C5a, which in turn can induce all classical signs of inflammation.

To prevent excessive activation, hyper-inflammation, and self-attack, the complement cascade is tightly regulated and controlled by complement regulatory proteins (CRegs). Main inhibitors within the fluid phase system are the C1 inhibitor (C1INH), C4-binding protein (C4bp), complement factor H, and complement factor I. On cellular surfaces there are also some potent surface-bound CRegs, e.g., complement receptor 1 (CR1, CD35), membrane cofactor protein (MCP, CD46), decay accelerating factor (DAF, CD55), and membrane inhibitor of reactive lysis (CD59). The balance between complement activation products and counteracting CRegs may be disturbed in many diseases, especially by the uncontrolled release of other potent proteases, e.g., during massive activation of the coagulation system or during inflammatory processes of the pancreas.

## Exocrine Pancreas and Complement as Central Players in Multiple Organ Dysfunction

It has been suggested that digestive enzymes (prematurely) released from the exocrine pancreas can enter the circulation *via* dysfunctional organ barriers. In the systemic circulation, these pancreas-derived proteases may cleave off cellular surface molecules and receptors and activate other proteases (24, 25), such as coagulation, fibrinolytic, and complement factors. The resulting “autodigestion” and dysregulation of important innate immune cascades reflect main pathophysiological features of MODS. Activated trypsin has been suggested to be ultimately responsible for MODS development, causing enhanced global organ permeability, blood exudate leakage, and coagulation dysfunction (26).

**Figure 2:**
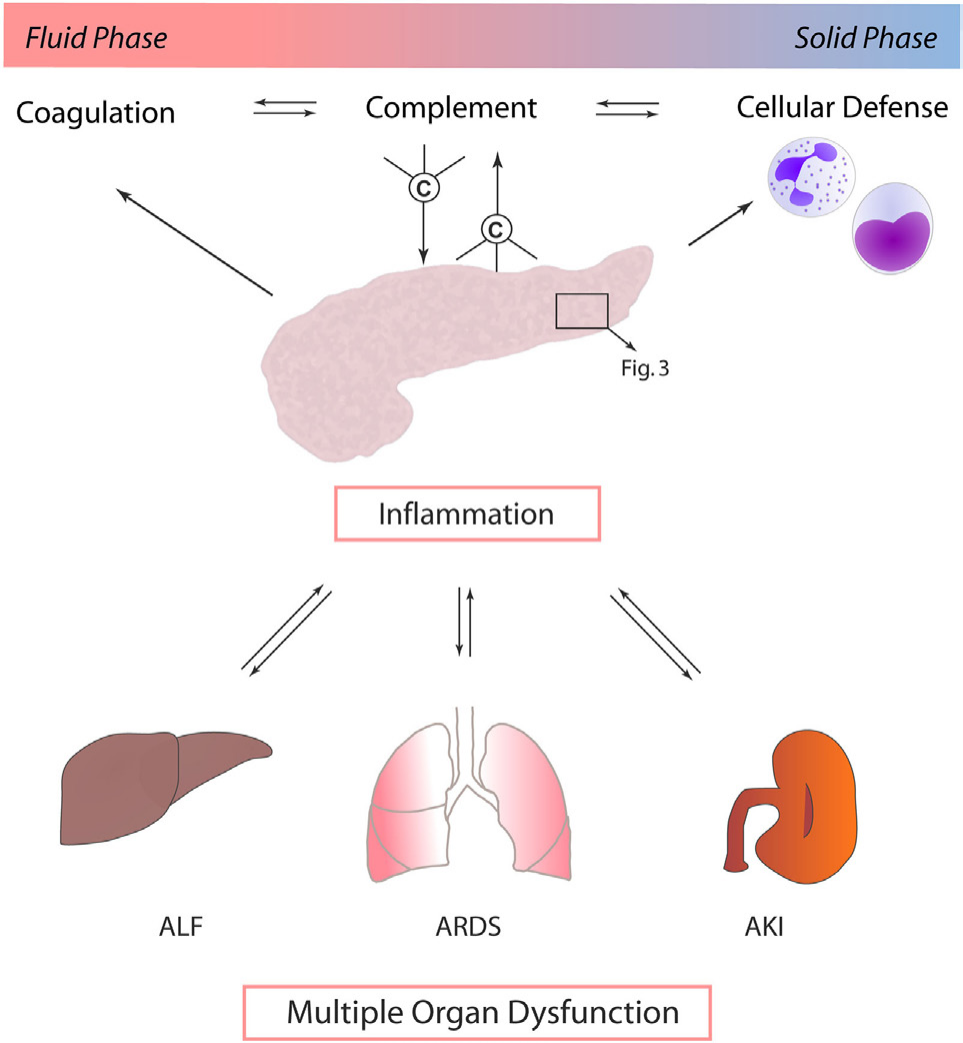
**Exocrine pancreas and complement as central players in multiple organ dysfunction**. In the context of acute pancreatitis, cross talk between complement, coagulation, and immune cells, such as neutrophil granulocytes and monocytes, drives multiple organ dysfunction. Systemic inflammatory response in turn mediates local organ damage and dysfunction. Abbreviations: ALF, acute liver failure; ARDS, acute respiratory distress syndrome; AKI, acute kidney injury.

Other mechanisms by which local pancreatic damage affects MODS have been proposed. In experimental pancreatitis, reduction of phosphorylation processes, impairment of the respiratory chain, and resulting severe mitochondrial dysfunction were found to facilitate further pancreatic damage. Beyond these local effects, mitochondrial function in the lungs and kidneys was also reduced early after induction of pancreatitis (27). A time-dependent onset of MODS (lung, kidney, heart, and liver) induced by experimental pancreatitis with associated acinar cell necrosis and systemic inflammatory response has been reported to involve neutrophil influx and extracellular regulated kinase (ERK) activation (28). Immune cell infiltration of lung tissue during experimental pancreatitis has been shown to be accompanied by enhanced expression of endothelial adhesion molecules, such as intercellular adhesion molecule-1 (ICAM-1) (29). Furthermore, when AP is complicated by infection, the excessive cytokine release and inflammatory response by macrophages further prime neutrophils for a “second attack” on remote organs (30) (Figure 2).

It is remarkable that most of the proposed mechanisms of pancreas-induced MODS have also been proposed in the context of systemic complement activation. It is of course questionable if complement activation *per se* is causative, contributive, or merely associated with MODS development. However, it is well established that the complement activation product C5a enhances ICAM-1 expression, effectively recruits and primes neutrophils, potentiates the cytokine release, activates multiple signaling pathways such as ERK, modulates the vascular tonus and micro-perfusion, and interacts with the coagulation system—only to mention some C5a effects (31, 32). Strikingly, *in vivo* blockade of C5a–C5aR interaction in sepsis-induced rodent MODS counteracted neutrophil dysfunction and protected cellular function on a multiple organ level, resulting in a significantly improved survival of MODS (7, 33, 34). Recently, a novel C5a-neutralizing mirror-image (l-)aptamer prevented organ failure and peritoneal barrier dysfunction in experimental sepsis (35), indicating C5a as a central player in MODS development. However, based on current literature, pancreas as a remote target of other severe organ dysfunction (such as ischemia/reperfusion injury of the kidney) has never been addressed in the context of complement activation. On the other hand, there is clear evidence that pancreatitis is associated with systemic complement activation (36), which *per se* may contribute to MODS development. In addition, the complement–coagulation cross talk (18, 20) certainly is crucially involved in progression of MODS and multiple organ failure (8, 34), with a potential role for the pancreas as a local and remote target. Concerning the spatial and temporal pattern of MODS development in the context of inflammatory pancreatic diseases, it is noteworthy that there is clinically and experimentally no characteristic pattern of organ failure onset. The order of affected organs rather seems to be dependent on the used experimental model and on the nature of the initiating pathogenic trigger (28, 37).

## Complement in the Pathophysiology of AP

**Figure 3:**
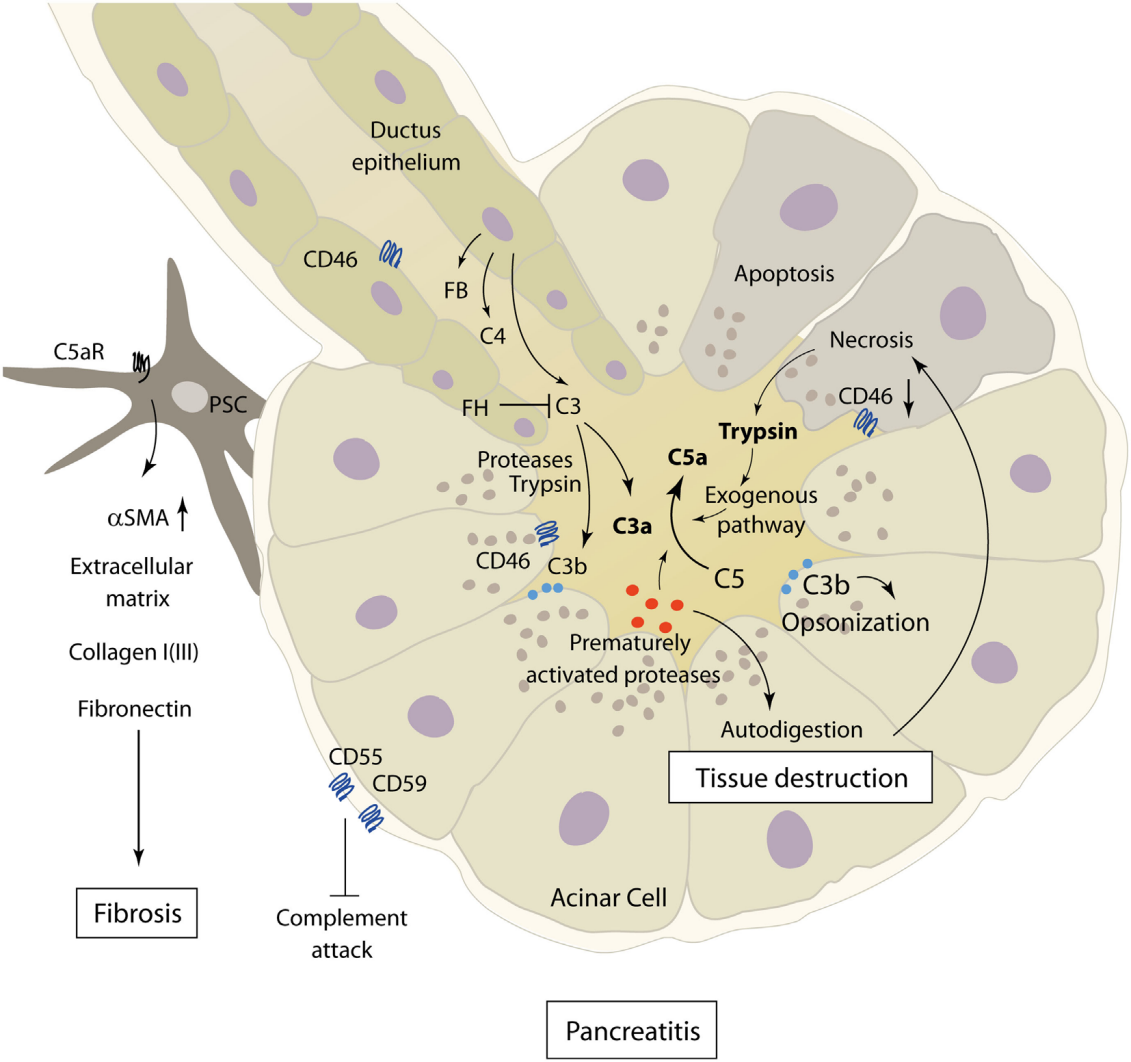
**Role of complement in the pathophysiology of acute and chronic pancreatitis**. The figure showcases some of the known complement–pancreas interactions as described in Sections “Complement in the Pathophysiology of Acute Pancreatitis” and “Implication of Complement in Chronic Pancreatitis and Autoimmune Pancreatitis.” Complement activation can induce acinar cell damage in early pancreatitis, which leads to autodigestion by pancreatic enzymes. Pancreatic proteases then in turn cleave and activate complement factors within the pancreas. Abbreviations: C3–C5, complement factor 3–5; C5aR, C5a receptor; FB, factor B; FH, factor H; PSC, pancreatic stellate cells; αSMA, alpha smooth muscle actin.

**Figure 4:**
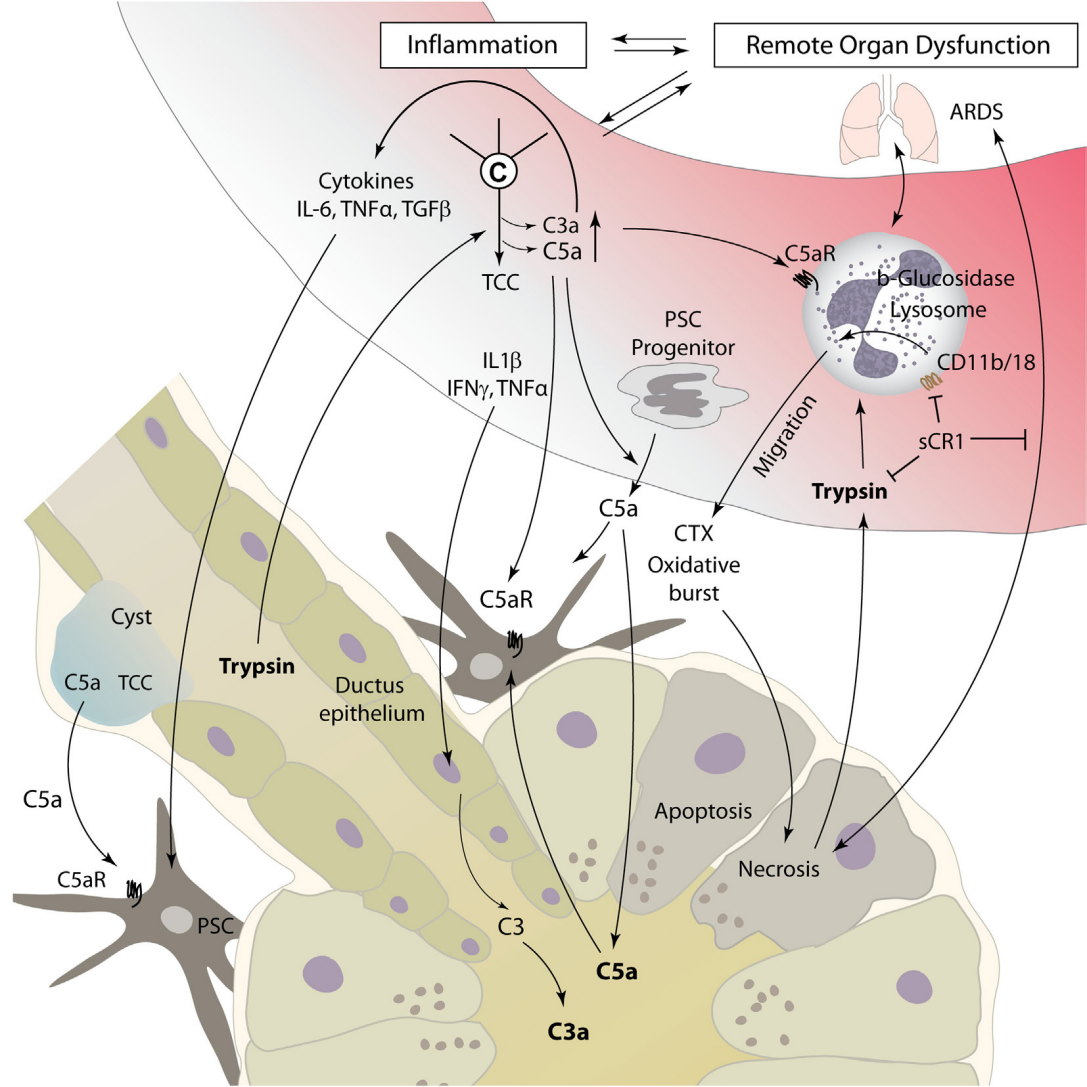
**Complement as a mediator of systemic inflammatory response during pancreatitis**. Local tissue destruction and complement activation caused by severe acute pancreatitis lead to a systemic inflammatory response, which can culminate in ARDS, shock, and multiple organ dysfunction. For details, see text. Abbreviations: ARDS, acute respiratory distress syndrome; C3–C5, complement factor 3–5; CTX, chemotaxis; IFN, interferon; IL, interleukin; sCR1, soluble complement receptor type 1; TGF, transforming growth factor; TNFα, tumor necrosis factor α; TCC, terminal complement complex.

Acute pancreatitis is a disease with extensive systemic effects, not only limited to its organ of origin. Intrapancreatic trypsinogen activation, mediated by cathepsin B, leads to autodigestion and promotes local organ damage (38). The ensuing destruction of exocrine parenchyma leads to a systemic inflammatory response with massive cytokine release and activation of the coagulation and kinin cascades. These effects can, in severe cases, lead to acute respiratory distress syndrome (ARDS), shock, and MODS. The pathomechanisms of complement involvement in local organ damage and systemic inflammatory response are outlined in Figures 3 and 4, respectively.

The complement system has long been implicated as an effector of local and distant inflammation in the pathogenesis and disease progression of AP. As early as 1975, it has been hypothesized that complement activation could be a factor initiating acinar cell damage in early necrotizing pancreatitis, which in turn leads to autodigestion by pancreatic enzymes (39–41). Decreased levels of serum complement components C3 and C4 during AP hinted at complement consumption and a potential role in driving local and systemic inflammation (42). While complement consumption in AP had been described by several authors at the time, one early study looking for C3 deposits in the pancreas during simultaneously falling C3 serum levels found no such stainable complement deposits in a murine model of dl-ethionine-induced AP (43, 44).

On the other hand, cleavage of C3 by the pancreatic protease trypsin with subsequent complement activation, a process only partly ameliorated by anti-proteolytic mechanisms in the process of excess trypsin release and activation, provides evidence of strong pancreas–complement interaction (45) (Figure 3). Complement activation seems to happen by both classical and alternative complement pathways, and the extent of changes in complement serum levels shows a correlation with the clinical severity of disease (36, 46, 47). Only severe necrotizing pancreatitis, but not mild edematous AP, in rats was accompanied by early complement activation with increased levels of C3a (48). Similar results linking decreasing complement hemolytic activity (CH50), acinar C3 deposits, and histological tissue damage could be found in a rodent model of AP (49). It is noteworthy that complement consumption in early AP can be accompanied by persistently lowered serum complement levels several weeks after the onset of symptoms (50).

In the clinical setting, C3a and sC5b–9 are elevated in the serum of patients with early AP and also show an excellent correlation with severity of disease, suggesting them as potential biomarkers for severe AP with high specificity and sensitivity (51). Patients who developed pancreatic pseudocysts as a result of AP showed changes in plasma complement activity and protease inhibitors similar to those seen in moderate to severe AP without pseudocysts (52). Pseudocyst fluid itself, as well as ascites of patients with AP, has been shown to contain high levels of C5a and terminal complement complex (TCC) (47).

*Vice versa*, in the absence of C5, C5-deficient mice develop significantly less pancreatic edema in the course of AP (53).

One recent study quantifying mannose-binding lectin (MBL) pathway effectors in AP patient sera found no significant correlation between levels of MBL, MBL-associated serine protease 2, and severity or mortality of disease. These findings suggest that the MBL pathway of complement activation only plays a minor role in AP pathogenesis (54). Fittingly, pancreatic MBL, isolated from pancreatic juice and identified as pancreatic elastase III, could not be shown to induce complement-mediated lysis, contrasting starkly with other known MBL isoforms (55).

The role of protease–anti-protease imbalance in this context has been extensively discussed, with contrasting results by different researchers, hinting at a more prominent role in local intraperitoneal trypsin activity as opposed to systemic (serum) activity. A low level of the anti-protease alpha-2-macroglobulin is an universally acknowledged predisposing factor for high trypsin activity (46, 56–59). Regarding protease–complement interactions, trypsin-induced C3 cleavage could be inhibited *in vitro* by high levels of pancreatic secretory trypsin inhibitor (60).

In addition to excessive complement cleavage and activation, loosening of physiologic inhibitory mechanisms controlling complement activity also plays a role in the pathophysiology of AP. The complement activation regulators CD46, CD55, and CD59, normally expressed on pancreatic acinar cells, are downregulated in sodium taurocholate-induced severe AP, and decreased expression is correlated with more severe necrosis. Downregulation of CD55 and CD59 could be alleviated by IL-4, with a positive effect on disease severity (61).

Interactions between humoral innate immunity and cellular immunity are decisive in the inflammatory response associated with AP. Different immune cells have been identified as important effectors in AP, especially macrophages and neutrophils (62). While data are scarce on macrophage/monocyte involvement in pancreatic complement modulation, the activation of neutrophil granulocytes by the complement system has been a focus of research. Especially, C5a is known to be a strong chemoattractant for neutrophil recruitment to damaged and inflamed tissue and can induce phagocytosis, release of cytokines, and oxidative burst with release of reactive oxygen species (63). Thus, C5a may represent a main driver for neutrophil migration to pancreatic tissue, as well as to lung tissue in AP-related ARDS (64) (Figure 4). Neutrophils incubated with serum from rats suffering from necrotizing AP showed increased expression of the adhesion molecule membrane attack complex (MAC)-1 (CD11b/18, complement receptor 3). In addition, injection of AP serum into lung tissue led to local neutrophil sequestration. Both effects were averted by complement inhibition with soluble complement receptor type 1 (sCR1) (65). In another rat model of acute severe/necrotizing and mild/edematous AP, inhibition of complement activation by administration of sCR1 decreased leukocyte–complement interaction and local and remote lung damage (48). Severe necrotizing AP seems to lead to a reduced expression of opsonin receptors, including complement receptor 3 on local peritoneal neutrophils, and enhanced expression on circulatory neutrophils. Nearly opposite changes were found in mild pancreatitis (66, 67). A 2001 study by Kyriakides et al. assessed the effects of total neutrophil and/or complement depletion in experimental AP. While local pancreas damage was not modified by either of those, neutropenia, but not complement depletion, showed a beneficial effect on remote organ injury and overall survival (68). This suggests that besides complement-mediated neutrophil activation, other mediators significantly contribute to the activation of cellular immune response during AP.

Contrasting anti-inflammatory effects of complement components have been reported in a 2001 study conducted by Bhatia et al. (69). In genetically modified mice lacking either C5 or C5a receptor, cerulein-induced pancreatitis was characterized by a distinctly more severe clinical progression as compared to wild-type mice, with aggravated local pancreas as well as remote lung injury. These findings hint at an anti-inflammatory role of C5a in AP. It should be noted that, when excessively generated, C5a eventually results in a functional paralysis of the neutrophil response (70) and thereby might represent a mechanism for inhibiting neutrophil-mediated pancreatic damage.

In conclusion, the role of the complement system in AP seems to be highly complex and is not yet fully understood. Opposing interactions concerning local and systemic inflammatory response have been proposed. Likely, both pro- and anti-inflammatory effects are conveyed by different key components within the complement cascade. On another note, the widespread usage of a number of different animal models, which vary considerably in their modes and mechanisms of AP induction, may have contributed to conflicting results.

## Implication of Complement in Chronic Pancreatitis (CP) and Autoimmune Pancreatitis (AIP)

In comparison to AP, not much research has been done on the role of complement in chronic or recurring pancreatitis as well as in AIP. The latter is sometimes associated with decreased serum complement levels, accompanied by high levels of circulating immune complexes during the active phase of disease, an effect hinting at complement activation *via* the classic pathway (71, 72). These immune complexes lead to the destruction of acinar cells and small- to medium-sized pancreatic ducts, as suggested by fluorescence staining for basal membrane deposits of C3c and immunoglobulins (73).

Concerning complement activity in CP, a recent study by Sendler et al. showcases the involvement of complement C5 in the development of fibrosis during CP. In C5-deficient mice, CP was induced by pancreatic duct ligation and cerulein injection or recurring cerulein injections alone. Mice lacking C5, as well as wild-type mice subjected to treatment with C5a-receptor antagonists, showed significantly reduced pancreatic fibrosis in later stages of disease. This seems to be a consequence of recruitment and activation of pancreatic stellate cells by C5a, shown *in vitro* and *in vivo*, leading to the production of extracellular matrix proteins (e.g., collagen type I, fibronectin, and αSMA) and fibrosis (74) (Figure 3). Interestingly, no major differences in disease severity and organ damage were noted during early stages of pancreatitis, which contradict the common understanding of complement involvement in AP.

## Potential Role of Complement in Pancreatic Tumorigenesis

Pancreatic cancer is among the most lethal common malignancies, as it is usually diagnosed at an advanced stage when curative therapies are no longer available. Pancreatic ductal adenocarcinomas (PDAC) constitute the overwhelming majority of pancreatic neoplasms. PDAC is characterized by extensive stromal desmoplasia, which promotes a hypovascular tumor microenvironment and immune evasion.

The inhibitory complement regulatory proteins CD46, CD55, and CD59 are known to be overexpressed in PDAC cell lines in a manner similar to other tumor entities (75, 76). This may contribute to immune evasion and impedes the potential of new immunotherapeutic strategies with monoclonal antibodies, which typically rely on complement-mediated cytotoxicity (77). Elevated serum levels of the inhibitory anti-proteases alpha-2-macroglobulin and C1INH constitute an additional mechanism of complement resistance in pancreatic cancer (78). Proteomic analysis of the cell line secretome confirmed inhibition of the complement system as a major functional alteration in PDAC (79). Furthermore, it has been shown that under inflammatory conditions, complement C3 secretion in PDAC cell lines was inhibited by TGF-β, a cytokine strongly involved in PDAC progression and tissue desmoplasia (80, 81).

Inhibition of the MBL pathway of complement activation has been described for a subgroup of PDAC patients. In a phase II clinical trial, treatment with ω-3-rich fatty acids in addition to chemotherapy restored MBL activity and improved patient survival (82).

The search for novel potential biomarkers for pancreatic cancer has led to several studies involving proteomics analysis of PDAC patient sera. Several authors describe elevated serum levels of complement components, such as C3, C3c, C5a, and C5b–9/MAC (83–86), as well as soluble iC3b, a complement product generated by the interaction with the abundantly expressed CRegs (87). Complement factor B has been proposed as a promising new serological biomarker for PDAC, supplementing CA 19-9 (88). However, biomarker performance, including C5, is severely impeded by obstructive jaundice, a pathology common in pancreatic cancer (89).

It is also notable that C4b1 expression in tumor tissue correlates with TNM staging (TNM Classification of Malignant Tumours, in which extent of disease is assessed by tumor size and infiltration, lymph node, and distant metastasis), with locally advanced and metastatic tumors showing higher expression levels (90).

To give one example for the relevance of complement in the setting of non-adenocarcinoma tumors of the pancreas, one recent study found a pro-tumorigenic role for C5 in neuroendocrine pancreatic tumors, with C5-deficient mice showing smaller primary tumors and fewer metastases. Likewise, in human NET samples, advanced tumors showed higher C5 expression than tumors with lower TNM staging (91).

Taken together, these findings suggest a balance of pro-inflammatory and anti-inflammatory complement modulation in PDAC. Pancreatic cancer is known to induce a pro-inflammatory microenvironment, promoting growth and metastasis, which involves the classical complement pathway. On the other hand, immune evasion of cancer cells themselves could be mediated by inhibition of MBL and upregulation of membrane-bound complement regulators.

## Therapeutic Complement Strategies in Pancreatic Diseases

Considering the aforementioned widespread implications of complement in the pathophysiology of a variety of different pathologies of the pancreas, only limited data exist on the possible therapeutic possibilities of targeted complement modulation in these diseases.

Combined treatment with C1 esterase inhibitor, an inhibitor of the classical complement pathway, and antithrombin III improved survival in a rodent model of severe AP. In contrast, the same treatment showed no significant benefit in mild edematous AP (92). Likewise, treatment with C1 esterase inhibitor improved the clinical outcome of severe AP after allogeneic hematopoietic stem cell transplantation in a case study of two pediatric patients. This therapeutic effect was mainly attributed to reduction of capillary leakage and subsequent cardiovascular stabilization (93). In a small-scale study of patients undergoing endoscopic papillosphincterotomy, C1INH prevented hyperamylasemia, a possible precursor of post-ERCP pancreatitis (94).

One other study found no beneficial effect of complement inhibition by sCR1 on cerulein-induced AP (95), while a later study with comparable methodology found an ameliorating effect on neutrophil accumulation in lung tissue, indicating therapeutic options for distant organ damage (96).

The positive effect of C5a-receptor blockage by the small molecule inhibitor W-54011 on fibrosis and disease progression in a rodent model of CP suggests therapeutical applicability in the chronic setting (74). One earlier study had seen similar results for the serine protease inhibitor camostat (97). Owning to the more generalized nature of inhibition, broad band serine protease inhibitors, although effective in complement inhibition, could be expected to carry more unwanted side effects than targeted complement inhibition. A recent meta-analysis of 17 clinical trials conducted over the last five decades could find no evidence for a beneficial effect of i.v. treatment with protease inhibitors on overall mortality in AP (98). Overall, no therapeutic strategies targeting complement in pancreatic diseases have been clinically established so far although complement modulation seems promising for the treatment of acute and CP.

## Future Avenues for Complement Research in Pancreatic Diseases

The data available clearly point to an important role of complement in induction and progression of acute and chronic inflammatory pancreatic diseases. However, it needs to be clarified whether the apparent Janus-faced nature of C5a in the development of pancreatitis is merely a question of concurrence with anti-inflammatory features during acute and pro-inflammatory features during chronic inflammation.

It is noteworthy that plasma anaphylatoxin levels correlate with the severity of pancreatitis (36, 46, 47). Therefore, it would be of utmost interest to determine whether this is only a reflection of systemic inflammation, or if the complement activation products are causative to induction and progression of pancreatitis and resulting complications. It seems that the role of local, uncanonical activation of complement by prematurely activated pancreatic proteases in the progression of pancreatitis has been rather underestimated. Thus, it is of importance to define which complement components can be locally produced by acinar cells and ductal epithelium and investigate the conditions of local complement cleavage and activation. In this regard, it is also surprising that no reports exist on the different anaphylatoxin receptor status (e.g., C3aR, C5aR1, and C5aR2) of pancreatic acinar cells. If detected, it would be of interest to characterize cellular signaling and functional consequences of these receptors.

Investigations of the role of complement in chronic inflammatory microenvironmental changes and cancer development are, if at all, *in statu nascendi*, but the subject certainly offers ample room for promising future research. In the light of recent findings linking C5 to fibrosis in CP (74), complement involvement in PDAC desmoplasmic stromal response could prove to be an interesting research topic. Furthermore, it has been shown in non-pancreatic tissues that complement proteins contribute to immune surveillance of malignant tumors (99). C5a in the tumor microenvironment notably enhanced tumor growth by inhibiting CD8+ T cell-mediated antitumor effects (100). On the other hand, iC3b opsonizes tumor surfaces and thereby supports tumor cell cytotoxicity, which is aided by a functional TCC (101). These contrasting findings highlight the need for further research into the role of complement components in pancreatic tumor development.

## Author Contributions

LB contributed to the conception, drafting, and writing of the manuscript, design and preparation of the figures, final approval, and agreement to any part of the work. SD contributed to the conception of the manuscript, critical revision of the work, design and preparation of the figures, final approval, and agreement to any part of the work. TS contributed to the conception of the manuscript, critical revision of the work, final approval, and agreement to any part of the work. MH-L contributed to the conception, design, and drafting of the manuscript, design of the figures, writing of the manuscript, final approval, and agreement to any part of the work.

## Conflict of Interest Statement

The authors declare that the research was conducted in the absence of any commercial or financial relationships that could be construed as a potential conflict of interest.
